# Emerging therapies against infections with *Pseudomonas aeruginosa*

**DOI:** 10.12688/f1000research.19509.1

**Published:** 2019-08-07

**Authors:** Burkhard Tümmler

**Affiliations:** 1 Clinical Research Group ‘Molecular Pathology of Cystic Fibrosis’ and ‘Pseudomonas Genomics’, Clinic for Pediatric Pneumology, Allergology and Neonatology, Hannover Medical School, Hannover, 30625, Germany; 2 Biomedical Research in Endstage and Obstructive Lung Disease (BREATH), German Center of Lung Disease, Hannover, 30625, Germany; 3 Cluster of Excellence RESIST (EXC 2155), Hannover Medical School, Hannover, 30625, Germany

**Keywords:** Pseudomonas aeruginosa, antibiotic, ß-lactam inhibitor, vaccine, phage therapy

## Abstract

Infections with *Pseudomonas aeruginosa *have been marked with the highest priority for surveillance and epidemiological research on the basis of parameters such as incidence, case fatality rates, chronicity of illness, available options for prevention and treatment, health-care utilization, and societal impact. *P. aeruginosa *is one of the six ESKAPE pathogens that are the major cause of nosocomial infections and are a global threat because of their capacity to become increasingly resistant to all available antibiotics. This review reports on current pre-clinical and clinical advances of anti-pseudomonal therapies in the fields of drug development, antimicrobial chemotherapy, vaccines, phage therapy, non-bactericidal pathoblockers, outer membrane sensitizers, and host defense reinforcement.

In humans, the aquatic gamma-proteobacterium *Pseudomonas aeruginosa* may cause multiple infections that vary from local to systemic and from benign to life-threatening. The management of the severe ocular ^1^ and burn ^2^ infections has made substantial progress during the last 20 years, but pneumonia and sepsis, particularly of ventilated patients in intensive care units (ICUs), are still burdened with high morbidity and lethality ^3, 4^. Chronic airway infections with *P. aeruginosa* are a major co-morbidity in patients with cystic fibrosis (CF) ^5^, bronchiectasis ^6^, or chronic obstructive pulmonary disease (COPD) ^7^.

Infections with *P. aeruginosa* have been marked with the highest priority for surveillance and epidemiological research on the basis of parameters such as incidence, case fatality rates, chronicity of illness, available options for prevention and treatment, health-care utilization, and societal impact ^8^. *P. aeruginosa* is one of the six ESKAPE pathogens that are the major cause of nosocomial infections in the US and are a threat all over the world because of their capacity to become increasingly resistant to all available antibiotics ^9^. *P. aeruginosa* is equipped with a lowly permeable outer membrane and multiple transport systems, rendering it naturally resistant to many antimicrobial agents ^10^. In addition to its intrinsic resistance common to all *P. aeruginosa*, the bacterium has the extraordinary capacity to develop resistance to nearly all available antimicrobials ^11^. The most common underlying mechanisms in multidrug-resistant (MDR) and extensively drug-resistant (XDR) *P. aeruginosa* are alterations in porin channels, efflux pumps, target modifications, and β-lactamases (for example, AmpC and carbapenemases) ^12, 13^. Resistance may be acquired by the selection of mutations in chromosomal genes or horizontal uptake of resistance determinants. Of particular concern are mobile genomic islands and integrons encoding carbapenemases or extended-spectrum β-lactamases (ESBLs) frequently co-transferred with aminoglycoside-modifying enzyme determinants ^14, 15^.

This review deals with the current approaches to develop new modes of anti-pseudomonal therapies. The decision of major pharmaceutical companies to exit antibacterial research has triggered the formation of non-profit alliances that support academia, clinicians, and industry in the development of novel antimicrobials. For example, Combating Antibiotic Resistant Bacteria (CARB-X) ( https://carb-x.org/) is funded by the US Department of Health and Human Services, the National Institutes of Health, the Wellcome Trust, the Bill & Melinda Gates Foundation, and Germany’s Federal Ministry of Education and Research. CARB-X is investing up to $550 million (USD) from 2016 to 2021 to accelerate the development of innovative antibiotics and other therapeutics, vaccines, and rapid diagnostics to address drug-resistant bacterial infections. Several companies have received funds from CARB-X to develop anti-pseudomonal agents, namely inhibitors of virulence factors and antibiotic potentiators ^16^. Support to develop novel drugs is also provided by the Innovative Medicines Initiative (IMI) funded jointly by the European Union and the European pharmaceutical industry. As described below, CARB-X and the IMI have been instrumental in speeding up the pre-clinical and clinical development of numerous anti-pseudomonal agents.

## Antibiotics

Antimicrobial chemotherapy is still the cornerstone of anti-pseudomonal treatment in clinical practice. *P. aeruginosa* is a naturally MDR organism, which may explain its success in becoming one of the most frequent nosocomial pathogens. Environmental *P. aeruginosa* strains are commonly susceptible to broad-spectrum penicillins and cephalosporins, aminoglycosides, monobactams, carbapenems, and fluoroquinolones. Since the 1980s, the intravenous combination therapy of piperacillin or ceftazidime with an aminoglycoside has been the standard of care to treat severe infections with *P. aeruginosa*, but the emergence of resistant organisms, particularly in the settings of intensive care or chronic persistence in vulnerable patient populations, has called for alternatives.

One strategy for the treatment of MDR *P. aeruginosa* has been the revival of colistin and polymyxin B, old drugs that had been abandoned for many years because of their significant toxicity and side effects. Within a few years of more active therapeutic use, a growing number of strains have meanwhile developed resistance against these last-line peptide antibiotics ^17, 18^. Mutations in various two-component systems activate the *arn* operon, which modifies the lipid A moiety of the lipopolysaccharide (LPS) through the addition of 4-amino-4-deoxy-L-arabinose, thereby rendering the bacterial cell resistant to the peptide antibiotic ^17, 18^.

An alternative strategy has been the development of molecules that overcome β-lactam antibiotic resistance. Two new cephalosporin-β-lactamase inhibitor combinations have recently been introduced into the clinic: ceftazidime-avibactam and ceftolozane-tazobactam ^19^. Avibactam readily inactivates the chromosomal β-lactamase of *P. aeruginosa* AmpC. Tazobactam is a less potent inhibitor of AmpC but this is compensated by the new antibiotic component in this combination, ceftolozane, which is only poorly hydrolyzed by AmpC.

At the time of this writing, more than 90% of *P. aeruginosa* isolates around the world seem to be susceptible to colistin, ceftazidime-avibactam, and ceftolozane-tazobactam ^15, 20– 23^. Resistance to the latter two antibiotic–antibiotic inhibitor combinations has been observed mainly in *P. aeruginosa* isolates that belong to the pandemic ST235 high-risk clone and carry novel isoforms of AmpD or ESBLs or both ^24, 25^.

Besides avibactam and tazobactam, other β-lactamase inhibitors—that is, relebactam ^26– 29^, zidebactam ^30, 31^, nacubactam ^32^, vaborbactam ^28^, VNRX-5133 ^33^, and AAI101 ^26^—are being tested in clinical trials.

An encouraging addition to the portfolio of anti-pseudomonal β *-*lactams is the siderophore cephalosporin cefiderocol ^34, 35^. Cefiderocol is structurally related to the cephalosporins ceftazidime and cefepime by sharing side chains that block recognition and inhibit hydrolysis by β-lactamases. The novelty resides in the extension of one side chain by a catechol 2-chloro-3,4-dihydroxybenzoic acid moiety. The catechol side chain enables ferric iron ion binding. The cefiderocol iron ion complex is recognized by active iron transport systems (such as PiuA) which transport cefiderocol across the outer membrane and to the periplasmic space ^36^. After dissociation of the complex, cefiderocol binds to penicillin binding proteins (PBP1a, PBP1b, PBP2, and PBP3) and inhibits peptidoglycan synthesis, causing cell death. Compared with the anti-pseudomonal agents that are currently available for use in humans, cefiderocol had the strongest activity against MDR *P. aeruginosa* strains. Of all β-lactams, cefiderocol has the most extended stability to hydrolysis by β-lactamases and its periplasmic entry via active iron transport systems overcomes β-lactam resistance associated with outer membrane permeability mutations in *P. aeruginosa* ^37^.

Cefiderocol is more potent *in vitro* against MDR *P. aeruginosa* than ceftazidime-avibactam and ceftolozane-tazobactam ^38^. Cefiderocol showed activity against AmpC-overproducing strains, low affinity for chromosomal AmpC β-lactamases, and a low propensity of temporal induction of AmpC β-lactamases of *P. aeruginosa* ^39^. Cefiderocol is active against carbapenem-non-susceptible isolates, including serine carbapenemase- and metallo-β-lactamase-producing strains ^40, 41^.

Carbapenem-resistant Gram-negative bacteria represent the highest priority for addressing global antibiotic resistance. Cefiderocol may address this problem—at least for some years to come. A recently completed phase II clinical trial demonstrated clinical efficacy and safety of intravenous cefiderocol compared with imipenem/cilastatin in patients with complicated urinary tract infections ^35^. Clinical trials of hospital-acquired pneumonia and carbapenem-resistant infections (ClinicalTrials.gov identifiers NCT02321800, NCT02714595, and NCT03032380) are ongoing.

Novel non-β-lactam antimicrobials have also been developed to target MDR organisms ^42^. Plazomicin is a sisomycin derivative that is unaffected by aminoglycoside-modifying enzymes. It was approved by the US Food and Drug Administration (FDA) for use in adults with complicated urinary tract infections. Its anti-pseudomonal activity is comparable to that of amikacin and less potent than that of tobramycin, indicating that this compound will probably not be very useful for the treatment of infections with *P. aeruginosa* ^42^. The same argument applies even more so to the tetracyclines eravacycline and omadacyline, which demonstrate antimicrobial activity against many Gram-positive and Gram-negative bacteria but are not active against *P. aeruginosa* ^42^. In contrast, two novel fluoroquinolones, finafloxacin ^43, 44^ and delafloxacin ^44, 45^, which (depending on pH) exert anti-pseudomonal activity equivalent to or higher than that of ciprofloxacin, have recently become available.

Besides these analogues of classes of antimicrobials well known for their basic chemical structure and mode of antimicrobial action, anti-pseudomonal drugs that aim at a new target are being developed. Murepavadin, a 14–amino acid synthetic peptidomimetic, is a first-in-class antibiotic targeting outer membrane protein ^46^. During the biogenesis of the outer membrane, new LPS molecules are transported from their site of assembly on the inner membrane to the outer membrane by seven LPS transport proteins (LptA–G). The complex formed between the outer membrane protein LptD and the lipoprotein LptE is responsible for transporting LPS from the periplasmic side of the outer membrane to its final location on the cell surface. Murepavadin inhibits the LPS transport protein LptD in *P. aeruginosa*. Murepavadin was proven to be a very potent antibiotic highly specific to *P. aeruginosa*, including carbapenemase producers and ceftolozane/tazobactam-resistant and colistin-resistant strains. Murepavadin (96.7% of isolates susceptible) was more active than colistin (93.6%), followed by ceftolozane/tazobactam (70.6%) and tobramycin (47.5%) ^47, 48^. Two clinical trials have been evaluating the efficacy and safety of murepavadin in treating lower respiratory tract infections caused by *P. aeruginosa* (suspected or confirmed) among patients with ventilation-associated pneumonia or bronchiectasis unrelated to CF (ClinicalTrials.gov identifiers NCT02096315 and NCT02096328, respectively). However, by July 17, 2019, the studies were stopped because an unexpectedly high frequency of renal failures had been observed in study participants who had received murepavadin. The development of an aerosolized formulation of murepavadin for a topical application will not be affected by this decision.

Murepavadin is a specific weapon against *P. aeruginosa*, which sets it apart from the large pipeline of natural and synthetic antimicrobial peptides that act against multiple taxa, including *P. aeruginosa*. Several novel peptides with broad antimicrobial activity—for example, DGL13K ^49^, Mel4 ^50^, melimine ^50^, cecropin B ^51^, LBP-2 ^52, 53^, Pse-T2 ^54^, 6K-F17 ^55^, MDP1 ^56^, and MDP2 ^56^—have recently been described.

Aerosolized anti-pseudomonal agents are the domain for the treatment of chronic airway infections of individuals with CF or bronchiectasis. Established options are the long-term inhalation with high-dose tobramycin ^57, 58^, colistin ^59– 61^, or aztreonam-lysine ^62– 64^. Emerging alternatives were the inhalation of liposomal amikacin ^65^ and, more recently, the inhalation with dry powder ^66, 67^ or liposomal ^68, 69^ ciprofloxacin or with liposomal levofloxacin ^70^. As described below, the clinical drug development programs had to face unforeseen obstacles unrelated to the proven anti-pseudomonal activity of the formulations *in vitro*.

Liposomal amikacin can penetrate within airway secretions and within *P. aeruginosa* biofilms, making it an attractive therapeutic option for chronic pulmonary infections. A phase II study with once-daily liposomal amikacin demonstrated acute tolerability, safety, biologic activity, and efficacy in CF patients with *P. aeruginosa* infection ^71^. However, apparently because of the results of a long-term rat inhalation carcinogenicity study, the FDA placed a clinical hold on the phase III clinical trials with this patient cohort by August 2017 and requested more safety data. Thirteen months later, the FDA approved inhalation with liposomal amikacin for the treatment of lung disease with *Mycobacterium avium* complex (MAC) in patients with refractory disease. Thus, at least until the time of this writing, the journey ended with a new therapy for MAC but not for *P. aeruginosa*.

Two phase III, double-blind, placebo-controlled trials—RESPIRE 1 ^67^ and RESPIRE 2 ^66^—examined the efficacy and safety of ciprofloxacin dry powder for inhalation (DPI) in patients with non-CF bronchiectasis who had experienced two or more exacerbations in the previous year and pre-defined bacteria in sputum, including *P. aeruginosa*, as a major pathogen. These two trials represent the largest clinical trial program ever conducted in bronchiectasis. RESPIRE 1 largely enrolled across Europe, North and South America, Australia, and Japan, whereas RESPIRE 2 focused on Asia and Eastern Europe. Patients received twice-daily ciprofloxacin DPI 32.5 mg or placebo in 14- or 28-day on/off treatment cycles for 48 weeks. The 14-day on/off treatment cycles in RESPIRE 1 significantly prolonged time to first exacerbation and reduced the frequency of exacerbations. The same trends were seen in the 14-day cycle in RESPIRE 2 and the 28-day cycles but did not achieve significance. When the data were pooled, an average 24% reduction in exacerbations was calculated. Experts who commented on the outcome of the trials concluded that aerosolized dry powder ciprofloxacin is most likely to be of benefit in selected patients with poorly controlled disease and very frequent exacerbations ^72^.

An alternative formulation to dry powder is the encapsulation of drug into liposomes. Two randomized, double-blind, placebo-controlled, phase 3 trials—ORBIT-3 and ORBIT-4—investigated the safety and efficacy of inhaled liposomal ciprofloxacin ^69^. The more than 500 study participants had had two or more pulmonary exacerbations treated with antibiotics in the prior 12 months, had non-CF bronchiectasis, and had a history of chronic *P. aeruginosa* lung infection. Compared with placebo, inhalation with liposomal ciprofloxacin led to a significantly longer median time to first pulmonary exacerbation in the ORBIT-4 but not in the ORBIT-3 trial. In a pooled analysis of data from the two trials, median times to first pulmonary exacerbation were 157 days in the placebo group and 222 days in the verum group, a non-statistically significant difference of 65 days (0.82, 95% confidence interval (CI) 0.65–1.02; *P* = 0.074).

For both the two RESPIRE and ORBIT trials, the results were not replicated. The discrepant outcome was attributed to differences in clinical practice and the vast ethnic, geographic, and endo-phenotypic heterogeneity of bronchiectasis ^72^. Future trials should address these differences across the globe and should thoroughly characterize the endo-phenotype of individual patients in order to identify the patient groups which benefit from specific modes of anti-pseudomonal treatment. Patient stratification within this highly heterogeneous group of patients makes sense in light of the experience with CF, which is a monogenic disorder of Caucasians. Most clinical studies on anti-pseudomonal chemotherapy in this more homogeneous patient population met their primary endpoints with smaller cohorts than the RESPIRE and ORBIT trials.

In randomized controlled trials, monotherapy with an aerosolized anti-pseudomonal drug has been proven to be an effective measure to suppress chronic airway infections with *P. aeruginosa* in CF *.* Comparable data on inhaled combination therapy are still missing. *P. aeruginosa* biofilms grown *in vitro* typically consist of a stalk-forming subpopulation situated in the deeper layer with low metabolic activity and a cap-forming subpopulation in the upper layer with metabolically active cells ^73^. Colistin preferentially kills the stalk subpopulation, whereas the cap-forming subpopulation is susceptible to the aminoglycoside tobramycin ^74^. Owing to this observation in biofilms as models for the sessile lifestyle of *P. aeruginosa* in CF airways, the sequential therapy with inhaled tobramycin and colistin was examined in an observational study with 41 CF patients with chronic *P. aeruginosa* infection ^75^. Treatment was well tolerated and significantly improved patients’ lung function. An alternative to colistin-tobramycin may be aztreonam-tobramycin. When biofilms were grown in flow cells, the alternation of tobramycin and aztreonam potentiated the bactericidal effect and the reduction in bacterial biomass ^76^.

Meanwhile, combination inhalation therapy has become routine in clinical practice, but besides the open-label exploratory study mentioned above, no clinical trials have yet been published. More clinical data about the efficacy of systemic combination therapy are available. For example, an 11-year single-center retrospective analysis of the treatment of *P. aeruginosa* bloodstream infections revealed that survival of patients receiving combination therapy (β-lactam-aminoglycoside or β *-*lactam-quinolone) was significantly higher than that of patients receiving β-lactam monotherapy ^77^. A recently published meta-analysis compared the outcome of empirical non-optimized double β-lactam combination therapy versus β *-*lactam plus aminoglycoside ^78^. In the 164 cases from 13 randomized clinical trials reported between 1972 and 1993, a response to *P. aeruginosa* was achieved in 58.5% for double β-lactam and 60.6% for β-lactam-aminoglycoside. The two regimens achieved similar clinical and microbiological responses, but nephrotoxicity and ototoxicity were significantly lower with double β *-*lactam combination therapy. These metadata are from a time period before broad-spectrum antibiotics were widely introduced into the clinic. Nevertheless, they tell us that double β-lactams may be a useful therapeutic option because synergy may arise from the complementary inactivation of sets of PBPs *.*

## Modulators of bacterial cell wall, transport, signaling, or virulence

Mucoid alginate-overproducing *P. aeruginosa* strains are a phenotypical hallmark of chronic airway infections in individuals with CF. Bacterial alginate is made of alternating blocks of mannuronate homooligomers and mannuronate-guluronate heterooligomers, whereas the algal alginate also contains guluronate homooligomers. Algal-derived alginate oligomers enriched in guluronate homooligomers (oligoG) reduce the viscosity of sticky biofilms and potentiate anti-bacterial and anti-fungal compounds. OligoG DPI is being tested in IMI-supported phase 2 clinical trials whether they improve lung function and respiratory symptoms in patients with CF.

The intrinsic multidrug resistance of *P. aeruginosa* is partly based on its low outer membrane permeability. By 1983, Vaara and Vaara introduced the concept of outer membrane–disorganizing sensitizers that make the outer membrane more permeable to amphiphilic and hydrophobic compounds ^79^. For example, the non-bactericidal polymyxin B nonapeptide sensitized *P. aeruginosa* strains 2- to 40-fold to ciprofloxacin, norfloxacin, and ofloxacin and 80- to 200-fold to the parent compound nalidixic acid, indicating that the higher anti-pseudomonal activity of fluoroquinolones compared with nalidixic acid was based not only on the more efficient inhibition of the DNA gyrase but also on a higher outer membrane permeability ^80^. Now more than 30 years after the first report of three outer membrane sensitizers, the approved anti-protozoal drug pentamidine ^81^ and the polymyxin B analogues SPR206 and SPR741 ^82, 83^ are in pre-clinical and clinical studies to re-fuel the anti-Pseudomonas pipeline.

*P. aeruginosa* uses quorum sensing, including the elastase (Las), rhamnolipid (Rhl), and Pseudomonas quinolone signal (PQS) systems, to regulate and coordinate population-wide group behaviors in infection processes like biofilm formation and the concerted secretion of virulence factors. Pathoblockers of the quorum sensing system abolish pathogenic features without affecting cell viability, providing the basis for a lower drug-induced selection pressure ^84– 87^. Potent inhibitors of all known quorum sensing systems have been identified, but none of the novel compounds such as NX-As-401 ( www.neembiotech.com) has yet made it into clinical trials. The exceptions are the well-known macrolide antibiotics. In the late 1980s, Japanese physicians reported that the chronic administration of erythromycin, clarithromycin, and azithromycin improved the clinical symptoms and prognosis of patients with chronic *P. aeruginosa* infections ^88^. Azithromycin does not kill *P. aeruginosa* but inhibits protein biosynthesis and quorum sensing ^89^. Azithromycin is now widely used for the treatment of chronic airway infections with *P. aeruginosa* in patients with COPD, bronchiectasis, or CF. Within the setting of the ICU, azithromycin showed a trend to prevent ventilation-associated pneumonia in intubated patients and significantly reduced the activation of quorum sensing–regulated virulence traits ^90^.

Iron metabolism is another highly topical target of anti-pseudomonal drug development. Gallium is an iron mimetic ^91– 95^. Ga ^3+^ has an ionic radius nearly identical to that of ferric iron Fe ^3+^ and hence can replace iron in Fe ^3+^-dependent biological systems. Unlike Fe ^3+^, Ga ^3+^ is not reduced under physiological conditions and thus inactivates iron-mediated redox cycling ^91– 93^. Gallium inhibited *P. aeruginosa* growth and biofilm formation and killed planktonic and biofilm bacteria *in vitro* ^91, 92, 94^ and increased survival in a murine infection model ^91, 92, 95^. Intravenous gallium treatment improved lung function in CF patients with chronic *P. aeruginosa* lung infection in a preliminary phase 1 clinical trial ^95^.

Neutralization of virulence effectors is another currently pursued approach to combat infections with *P. aeruginosa.* Some programs are supported by the CARB-X alliance. For example, inhibitors are developed against the *P. aeruginosa* LasB elastase ( https://antabio.com/programs), thereby targeting the bacterium’s ability to evade the immune system and cause disease and, when given alongside antibiotics, helping to clear *P. aeruginosa* infections. Other programs have focused on the machinery and virulence effectors of the type III secretion system. Phenoxyacetamide inhibitors target the needle protein PscF that delivers the virulence effectors into the host cell ^96, 97^. Alternatively, monoclonal antibodies were generated against the PcrV protein that forms the tip of the injectosome complex ^98^. Intravenous KB001-A, an anti-PcrV PEGylated monoclonal antibody fragment, showed limited efficacy in CF patients infected with *P. aeruginosa* ^99^. The repeated administration of KB001-A over 16 weeks was associated with a small improvement of lung function and decrease of sputum inflammatory markers but did not prolong the time-to-need for antibiotics for worsening respiratory signs and symptoms ^99^. Bispecific antibodies that block multiple evasion and subversion mechanisms in tandem may be more efficacious ^100^. In a murine bacteremic model of *P. aeruginosa* infection, the bispecific therapeutic antibody MEDI3902, targeting PcrV and the Psl exopolysaccharide, was shown to efficiently enhance neutrophil uptake, phagosome acidification, and bacterial killing ^101^. After completion of a phase 1 study ^102^, passive immunization with MEDI3902 (renamed Gremubamab) is currently in phase 2b development for prevention of nosocomial *P. aeruginosa* pneumonia in patients undergoing mechanical ventilation (EVADE study funded by the IMI).

## Novel formulations for anti-pseudomonal drug delivery

Impaired penetration of antimicrobials through bacterial biofilms is one of the reasons for the failure of anti-pseudomonal therapy of burn wounds and chronic lung infections. Encapsulation of antimicrobials in nanocarriers may facilitate drug diffusion within the sticky biofilm matrix, protect the drug from unwanted degradation, confer controlled drug release, and increase uptake by the drug target. Anti-pseudomonal drugs such as ciprofloxacin ^103– 105^, meropenem ^106^, tobramycin ^107, 108^, gentamicin ^109^, or amikacin ^110^ were encapsulated into liposomes or loaded into nanoparticles. The drug delivery systems were diverse in chemical nature and include anionic liposomes ^105, 106, 109^, poly(lactic-co-glycolic) acid nanoparticles ^110, 111^, water-soluble chitosan oligosaccharide conjugates ^112^, oil-in-water cross-linked polymeric nanocomposites ^113^, graphen-oxide conjugates ^107^, or solid lipid nanoparticles ^114^, to name just a few. Alternatively, dry powders ^103, 104, 108, 115^ or hydrogels ^116– 119^ were formulated or wound dressings were coated with a topical antimicrobial such as silver oxynitrate ^120^. Irrespective of the chosen formulation, most published articles report that their formulation penetrates through mucus and biofilms, is more effective than the antimicrobial alone to eradicate biofilm formation, and mitigates infection and disease progression.

## Vaccines

The provision of an effective vaccine to protect patient populations at risk from an infection with *P. aeruginosa* has been on the agenda of Pseudomonas researchers for many decades, but there are no licensed vaccines at present. In the 1990s, the Swiss Serum and Vaccine Institute developed an octavalent *P. aeruginosa* O-polysaccharide-toxin A conjugate vaccine for immunization of healthy *P. aeruginosa*–negative patients with CF ^121^. The persistence of high-affinity antibodies among immunized patients correlated with a significantly lower rate of infection after 4 to 6 years of observation. The vaccine was well received by the European CF community. Patients at my CF center regularly travelled to Bern, Switzerland, to receive boosters every two to three years until the stock was used up. A few years later, Döring *et al*. conducted a double-blind, placebo-controlled, multicenter trial with a flagella vaccine demonstrating that active immunization of patients with CF lowers the risk for infection with *P. aeruginosa* ^122^. The third approach was the 20-year-long development of vaccines based on OprF-OprI outer membrane fusion proteins as antigen ^123^. In the last pilot study, published in 2010, human volunteers were vaccinated with a systemic, nasal, or oral live vaccine based on attenuated live Salmonella (strains CVD908 and Ty21a), followed by a systemic booster ^124^. Systemic and mucosal vaccines induced a comparable rise of serum antibody titers, but only nasal and oral vaccinations elicited a significant rise of IgA and IgG antibodies in the lower airways. At that time, the authors concluded that nasal and oral OprF-OprI vaccines were promising candidates for development of anti-pseudomonal immunization through inducing a specific antibody response in the lung.

These old data, including small clinical trials on burns and CF, provided evidence that a vaccine could be an effective measure to prevent infections with *P. aeruginosa.* Hence, the recombinant OprF-OprI vaccine was tested in a randomized, placebo-controlled, double-blind phase II/III study ^125^, which was conducted in 800 mechanically ventilated ICU patients at 52 trial sites in six European countries. Patients were vaccinated twice with either the *P. aeruginosa* vaccine candidate or a placebo at a 7-day interval in conjunction with standard-of-care treatments for ICU patients. Although the trial confirmed good immunogenicity and an acceptable safety profile of the vaccine candidate, the primary endpoint of the phase II/III trial was not met. Therefore, findings from a previous phase II study that had shown a strong reduction in all-cause mortality were not confirmed.

The outcome of this largest-ever trial performed on a Pseudomonas vaccine was disappointing. Nevertheless, there are encouraging new data on other antigens. For example, a live *aroA-aroB* attenuated *Salmonella* vaccine that uses a fusion between the *P. aeruginosa* type III secretion antigen PcrV expressed under the control of the sseA promoter and the *S. enterica* type III secretion effector protein SseJ has been constructed ^126^. Compared with control mice, mice immunized with attenuated *Salmonella* expressing this fusion had lower serum levels of pro-inflammatory cytokines and reduced bacterial loads in the spleen and lungs after *P. aeruginosa* infection. Importantly, in this model, immunized mice also showed significantly enhanced survival. Another novel strategy is the design of live-attenuated whole cell vaccines based on D-glutamate auxotrophy ^127^. The enzyme glutamate racemase MurI converts the amino acid L-glutamate into its enantiomer D-glutamate, which is an essential component of peptidoglycan. In-frame deletion of the *murI* gene generated a live-attenuated *P. aeruginosa* auxotrophic strain that, upon local or systemic administration, triggered appropriate cellular immune responses and production of specific and cross-reactive antibodies in the vaccinated murine hosts and conferred long-term survival against lethal infections with *P. aeruginosa* but, on the other hand, was rapidly eliminated from the host without causing disease. Other groups showed protection in murine infection models by using the iron acquisition protein HitA ^128^, PA5340 combined with PA3526-MotY ^129^, PcrV with CpG oligodeoxynucleotide ^130^, or the pilus proteins PilQ and PilA ^131^ as vaccine antigens.

## Phage therapy

Given that antibiotic resistance is an increasing threat not only to human health but also to the production of food and to sustainable development, phage therapy is regaining interest as an alternative or addition to antibiotic therapy for the treatment of bacterial infections ^132^. Phage therapy was abandoned in many countries with the advent of antibiotic therapy but has been continually developed in Eastern European countries with centers in Warsaw, Poland, and Tbilisi, Georgia ^133^. Shotgun metagenome sequencing revealed that the phage cocktails sold in pharmacies in Georgia and Russia contained anti-pseudomonal phages ^134^. A few case reports from Belgium and the US communicated the successful treatment of infections with MDR *P. aeruginosa* ^135, 136^.

Early this year, the outcome of the first clinical study on phage therapy was reported ^137^. The study, conducted as a randomized controlled double-blind trial, compared the tolerability and efficacy of a cocktail of lytic anti- *P. aeruginosa* bacteriophages with standard of care for patients with burns. The primary endpoint—the median time to sustained reduction in bacterial burden—was reached in 47 hours in the standard-of-care group (hazard ratio 0.29, 95% CI 0.10–0.79; *P* = 0.018) versus 144 hours (95% CI 48–not reached) in the group that received the phages. The finding that a standardized phage cocktail decreased bacterial burden in burn wounds more slowly than the standard of care is a strong indication that phage cocktails of fixed composition could unfavorably interfere with the evolutionary race between phage and bacterium by selecting phage resistance in the heterogeneous bacterial populations that vary from patient to patient. The personalized approach of choosing phages that specifically target the Pseudomonas bacteria in the individual host habitat may be more effective, although it will require rethinking of the regulatory agencies.

Research is very active in the pre-clinical arena. Practical themes are the setup of efficacious and safe antibacterial phage cocktails, the design of clever infection models, and the development of phages as adjuvants of antibiotic therapy. More importantly, if we want to make phage therapy a success, we need an in-depth understanding of how the mutual evolutionary race of attack and resistance between phage and bacterium takes place. Phages are, in principle, a smart anti-pseudomonal weapon. They specifically target a narrow spectrum of hosts, self-amplify, kill antibiotic-resistant strains, and have limited immunological effects in humans. However, it will not be a global anti-pseudomonal weapon. During chronic infection, *P. aeruginosa* may modify or delete all of its phage receptors. The author noticed that the majority of *P. aeruginosa* clones that persisted for five years or more in a CF lung had become pan-resistant to phages. **

## Hygienic measures

*P. aeruginosa* is responsible for a wide range of acquired infections in critically ill patients. Microbiological monitoring according to Clinical and Laboratory Standards Institute standards, antimicrobial stewardship, and infection control programs, including environmental cleaning and disinfection, hand hygiene, and education of personnel, have been demonstrated to prevent the development of resistance in *P. aeruginosa* ^138^. Prophylactic antibiotic days and inadequate empiric antibiotic therapy are independent major risk factors for the emergence of MDR ventilator-associated pneumonia in the ICU ^139^. Thus, prolonged exposure to unnecessary antibiotics should be avoided.

In the hospital setting, *P. aeruginosa* may contaminate sanitary facilities, humid medical devices, aqueous solutions, soaps, and detergents ^140^. For example, recently published case reports identified sinks or flexible endoscopes as reservoirs for nosocomial transmission of *P. aeruginosa* ^141, 142^. Sinks in hospitals are regularly contaminated with *P. aeruginosa*. Opening of water taps generates aerosols containing *P. aeruginosa* sink organisms that contaminate the faucet and hands during hand washing ^140^. Installation of filters under all water faucets has been shown to prevent bacterial contamination of tap water ^143^.

In the context of CF, patient-to-patient transmissions of *P. aeruginosa* were reported from CF clinics, summer camps, and rehabilitation centers ^5, 144^. Transmissible epidemic clones spread at CF clinics in Australia, Canada, Denmark, The Netherlands, and the UK ^5^. Hence, infection prevention and control practices have been introduced into CF clinics encompassing education, temporal separation of *P. aeruginosa*–positive and *P. aeruginosa*–negative patients, hand and cough hygiene, and cleaning and disinfection of equipment ^145^. Retrospective and prospective observational studies performed after the introduction of cohort segregation have demonstrated decreases in the numbers of prevalent and incident cases of epidemic *P. aeruginosa* infections ^5^.

## Enhancement of host defense

Active immunization of vulnerable patient groups is the classic approach to prevent microbial infection. But in real-life situations such as an acute illness requiring hospitalization, the time span to mount protective antibody titers may be too long to be clinically meaningful.

In the ICU, treatment with antibiotics often is live-saving but is also a major risk factor for subsequent nosocomial lung infection with *P. aeruginosa*. A recent study by Robak *et al*. demonstrates that the ICU patient’s susceptibility to secondary Pseudomonas infection is caused by antibiotic-associated secondary IgA deficiency ^146^. Depletion of the resident microbiota by broad-spectrum antibiotic treatment inhibits the stimulation of pulmonary IgA production mediated by microbiota-dependent activation of Toll-like receptors and the tumor necrosis factor (TNF) family cytokine APRIL (a proliferation-inducing ligand). If antibiotic-pretreated mice received IgA by the nasal route, their antibacterial defense against *P. aeruginosa* was partially restored. The authors propose that ICU patients on broad-spectrum antimicrobial therapy may benefit from prophylactic or therapeutic pulmonary IgA administration or both.

Cell-based treatment is another emerging option to target airway infections with *P. aeruginosa* ^147^. Therapeutic phagocytes such as macrophages can be produced from induced pluripotent stem cells (iPSCs) in industry-compatible, stirred-tank bioreactors. iPSC macrophages rescued mice from *P. aeruginosa*-mediated acute infections of the lower respiratory tract within 4 to 8 hours after intra-pulmonary transplantation and reduced bacterial load ^147^. This type of cell therapy may become an option for the treatment of congenital or acquired immune deficiency.

## Conclusions

The ESKAPE pathogens are the tip of the iceberg of the global antibiotic crisis. Many regions in the world now face infections with *P. aeruginosa* that is colistin- or carbapenem-resistant or both. Fortunately, the traditional approach to develop derivatives of validated scaffolds is still promising. The novel β-lactam inhibitors and siderophore cephalosporin are active against almost all current *P. aeruginosa*. The modification and combination of lead modules that tackle well-characterized bacterial targets constitute a rather safe approach to come up with an antimicrobial that will show efficacy and safety in clinical trials. The development of compounds against novel targets should be more rewarding in the long run. However, as we now experience with the currently most potent anti-pseudomonal agent, murepavadin ^47, 48^, the risk of off-target side effects is high and the compound may fail in clinical trials.

Pathoblockers have finally come of age ^81, 84– 87^. Ten to forty years after the proof-of-principle experiments showing that small molecules may reduce fitness or virulence of *P. aeruginosa* without being bactericidal themselves were published ^79, 98, 148^, the first sensitizers are now being examined in clinical studies ^83^. For example, the iron biomimetic gallium attacks *P. aeruginosa* at its metabolic achilles’ heel ^91– 95^. The outcome of the first clinical study is encouraging ^95^; however, we still do not know whether gallium will drive *P. aeruginosa* cells into an iron deficiency status that may promote the adverse production of virulence factors ^149^.

Given the threat of bacteria that are pan-resistant to the patient’s *P. aeruginosa* isolates, phage therapy is re-emerging as an attractive alternative to treat infections with *P. aeruginosa.* The outcome of the first high-standard clinical trial, published earlier this year, taught us that predetermined phage cocktails will probably not be the solution ^137^. Some *P. aeruginosa* strains will not be susceptible or will rapidly become resistant to the administered phages. To make phage therapy globally efficacious, we need a personalized approach as was recently demonstrated for a life-threatening infection with *Mycobacterium abscessus* ^150^. Phage cocktails should be formulated on a case-by-case basis to specifically target of phage therapy. However, phage therapy will leave its niche only if the regulatory agencies change the legal rules and permit personalized medicine on a large scale.

The development of antimicrobials is the classic approach to fight infections with *P. aeruginosa*. Only recently, the scientific community started to adopt the concept that the enhancement of host defense may be a promising alternative to conquer a nosocomial pathogen that causes severe infections in vulnerable populations but is more or less innocent for the healthy immunocompetent host. Relying on clinical experience in the ICU of the often disastrous course of secondary Pseudomonas pneumonias, researchers are becoming aware of the importance of the interplay between immune status and microbiome to contain this nosocomial pathogen ^146^. The enhancement of innate and adaptive immunity is a promising approach to vanquish MDR and XDR *P. aeruginosa.* Bispecific therapeutic antibodies ^101, 102^ and local transfer of isogenic iPSC-derived immune cells ^147^ could become the weapons of the future to prevent the fatal outcome of *P. aeruginosa* pneumonia and sepsis in ICU patients.

## Abbreviations

CARB-X, Combating Antibiotic Resistant Bacteria; CF, cystic fibrosis; CI, confidence interval; COPD, chronic obstructive pulmonary disease; DPI, dry powder for inhalation; ESBL, extended-spectrum β-lactamase; FDA, US Food and Drug Administration; ICU, intensive care unit; IMI, Innovative Medicines Initiative; iPSC, induced pluripotent stem cell; Las, elastase; LPS, lipopolysaccharide; Lpt, lipopolysaccharide transporting protein; MAC, *Mycobacterium avium* complex; MDR, multidrug-resistant; OligoG, oligomers enriched in guluronate homooligomers; PBP, penicillin binding protein; XDR, extensively drug resistant
